# Childhood anemia in Rural Haiti: the potential role of community health workers

**DOI:** 10.1186/s41256-016-0022-7

**Published:** 2017-01-23

**Authors:** Marie N. Séraphin, Chen Xinguang, Mohamed Ag Ayoya, Ismael Ngnie-Teta, Ellen Boldon, Aissa Mamadoultaibou, Jean Ernst Saint-Fleur, Inobert Pierre

**Affiliations:** 1 Department of Medicine, Division of Infectious Diseases and Global Medicine, University of Florida, 2055 Mowry Road, Suite 250, PO Box 103600, Gainesville, FL 32610-3600 USA; 2 Department of Epidemiology, College of Public Health and Health Professions and the College of Medicine, University of Florida, Gainesville, FL USA; 3 UNICEF Country Office, 125 Rue Faubert, Petionville, Port-au-Prince Haiti; 4 St. Boniface Haïti Foundation, 12 Rue E. Guello, Fond des Blancs, Haiti

**Keywords:** Iron Deficiency Anemia, Community Health Worker, Structural Equation Modeling Model, Maternal Knowledge, Childhood Anemia

## Abstract

**Background:**

Childhood iron deficiency anemia (IDA) is an important contributor to under-five mortality in the developing world. There is evidence that Community Health Worker (CHW) delivered programs to increase maternal knowledge of child health practices may decrease childhood IDA. This study reports findings on the association between a long standing CHW intervention and childhood anemia status in rural Haiti.

**Methods:**

Using structural equations and mediation analyses on data from a household-based survey of 621 mother/child dyads, we tested the hypothesis that CHW would have a direct positive effect on maternal knowledge and an indirect effect on childhood anemia in rural Haiti.

**Results:**

CHW contact was significantly associated with maternal knowledge of key child health practices (β = 0.193, SE = 0.058, *p* = 0.001). However, knowledge was not associated with childhood anemia (β = -0.008, SE = 0.009, *p* = 0.382). Maternal knowledge categories significantly affected by CHW contact included diarrheal prevention knowledge (β = 0.111, SE = 0.045, *p* = 0.013) and signs of malnutrition (β = 0.217, SE = 0.071, *p* = 0.002). There was no significant association with knowledge of vitamin A and iron rich foods (β = 0.057, SE = 0.032, *p* = 0.074), which is the intervention most likely to impact childhood anemia. In all path models tested, we identified the control variables low household socio-economic status, mothers’ anemia status, and child’s age less than 24 months as significant predictors of childhood anemia.

**Conclusions:**

CHWs delivered interventions are associated with improved maternal knowledge of child health practices in rural Haiti; however, this knowledge is not associated with improved childhood anemia. Concurrently with CHW-delivered programs, interventions household poverty are implied to impact childhood health outcomes in resource poor settings.

**Electronic supplementary material:**

The online version of this article (doi:10.1186/s41256-016-0022-7) contains supplementary material, which is available to authorized users.

## Background

Iron deficiency (ID) is the most prevalent nutritional deficiency worldwide [1]. Iron deficiency anemia (IDA), a manifestation of chronic ID is a global public health challenge, especially among children less than 5 years old in the developing world [2]. The World Health Organization (WHO) estimates that about 43% of children 6 to 59 months worldwide are anemic [1]. In 2013, 6.3 million children under the age of 5 years died in low and middle income countries; 45% of these deaths were attributable to nutrition-related factors, such as chronic under-nutrition, stunted growth, and IDA [3]. In addition to increased risk of under-five mortality, childhood anemia is associated with growth retardation, impaired motor and cognitive development [4, 5]. Nutrition-related risk factors for childhood IDA are easily preventable if mothers exclusively breastfeed their babies, properly introduce complementary food and know how to prevent and treat diarrheal diseases [3]. However, the prevalence and adoption of these live-saving practices are low [6], potentially due to a lack of maternal health literacy [7]. Several studies have identified maternal education as the most salient predictor of child health [8–11]. Concurrently, there is strong evidence to support that interventions aimed at increasing maternal knowledge, independent of maternal education, have the potential to have a positive effect on child nutritional outcomes [12–15]. In low resource settings, health education messages delivered by community health workers (CHWs) are among some of the interventions used to increase maternal knowledge and promote child health outcomes [16–19].

Haiti has used CHWs since the early 1980s for the promotion of vaccination, behavioral change communication, maternal and child health, nutrition and weight monitoring and later HIV and tuberculosis treatment support and supervision [20]. However, evidence on the effectiveness of such programs are limited. In Southern Haiti, the St. Boniface Haiti Foundation (SBHF) relies heavily on a network of 72 CHWs to carry out community-based primary health care programs in Fond des Blancs and Villa, two predominantly rural regions of approximately 65,000 inhabitants. SBHF CHWs, also known as *Collaborateurs Volontaires* or *Colvols* conduct monthly vaccination rally posts where children accompanied by their mothers/caregivers receive vaccination, weight monitoring and behavior change communication. The SBHF community outreach program is modeled after a similar program started in the early 1980s by Hôpital Albert Schweitzer in Artibonite, Haiti [21]. Initially trained by the Catholic Relief Services, SBHF *Colvols* are teachers, religious and political leaders in their community. Each *Colvol* maintains a registry with the names of all children under 5 years and pregnant women in their respective communities and reports on the health status of these groups to SBHF Community Health staff on a monthly basis. SHBF *Colvols* receive annual refresher training from SHBF nurses trained in Community Health. SBHF has been servicing the Southern region of Haiti for over 30 years and CHWs are an integral part of their community outreach activities.

In 2011, with a grant from UNICEF Haiti, SBHF carried out an evaluation of their community-based nutrition, maternal and child health programs in preparation for implementation of a project to improve these outcomes in the population. In Haiti the prevalence of anemia among children 6 to 59 months is 65.0% [22]. In addition, urban children are more likely to be anemic compared to rural Haitian children, 66.0% compared to 64.5% [22]. Findings from the evaluation showed that in the predominantly rural SBHF catchment area, 38.8% of children are anemic, a significantly lower prevalence than recorded elsewhere in Haiti [23]. The evaluation also presented an opportunity to measure the effect of the long standing CHW intervention on maternal knowledge of key child health practices and potential impact on childhood IDA in the region. Using structural equations and mediation analyses, we tested the hypothesis that contact with CHWs would have a direct effect on maternal knowledge and an indirect effect through maternal knowledge to reduce childhood IDA in the SBHF catchment area.

## Methods

### Participants

We analyzed data from a cross-sectional household based survey conducted from October to November 2011 in a predominantly rural region of about 65,000 inhabitants situated in the southern region of Haiti. The survey design and procedures have been described previously [23, 24]. Briefly, a total of 800 households were selected for the survey using a two-stage sampling methodology. First, 30 out of 69 villages were selected applying probability proportional to size. Second, 30 households within each village were selected. Households had to have a child under the age of 5 years to be included in the survey. Overall, 828 women of child bearing age (15–49 years) and their youngest child under the age of 5 years were recruited for the study. A close-ended, pre-coded questionnaire was used to gather information on household characteristics, food security, water sanitation and hygiene (WASH), women and newborn health, breastfeeding and complementary feeding, care for illnesses such as diarrhea and fever, anemia and anthropometry for both the index child and mother/caretaker.

### Measures

#### Community Health Worker Contact (CHW)

Five indicators measuring the latent construct CHW contact (Cronbach alpha = 0.72) was assessed with data derived from the responses to the question: “Did you receive the following information from a CHW? a) on child weight/growth, b) on exclusive breast feeding, c) on complementary feeding, d) on family planning, e) on child caring”. Each item was modeled as a binary categorical variable and set to load on the latent factor CHW which captured the promotional health education mothers reported to have received. CHW often meet community members in group settings at vaccination or health rally posts. Although it is likely that some mothers had more contact than others, in these analyses we assumed that all mothers received equal contact to a CHW.

#### Maternal Knowledge

SBHF Colvols communicate child health practices around exclusive breastfeeding (EBF), diarrheal disease prevention, malnutrition prevention, and childhood nutrition in the form of small group discussions at the monthly rally posts. We measured maternal knowledge of diarrheal disease prevention (Cronbach alpha = 0.99) using the multiple-choice question: “*How can diarrhea be prevented?* Maternal knowledge of nutrition was assessed using a question asking mothers to name foods rich in Vitamin A and Iron (Cronbach alpha = 0.98). Finally, knowledge of malnutrition signs in their child (Cronbach alpha = 0.99) was measured from the multiple-choice question: “*can you name the signs that a child with malnutrition may have?*” The full list of questionnaire items used in this study is presented in Annex (Additional file 1: Appendix A). Interviewers were instructed to probe the mothers to name as many options as possible. Mean scores for each of the three knowledge measures were computed such that high scores indicated more knowledge.

#### Childhood Anemia Status

Mother and child dyads’ hemoglobin levels were measured using the HemoCue machine according to a standardized protocol. Anemia was defined as hemoglobin levels less than 11 g/dL for children and pregnant women and levels < 12 g/dL for non-pregnant women. Childhood IDA was not objectively confirmed with serum ferritin and transferrin receptors measurements. We used childhood anemia as a proxy measure for IDA, since ID is the major cause of anemia in the developing world [2].

### Statistical Analyses

**Fig. 1:**
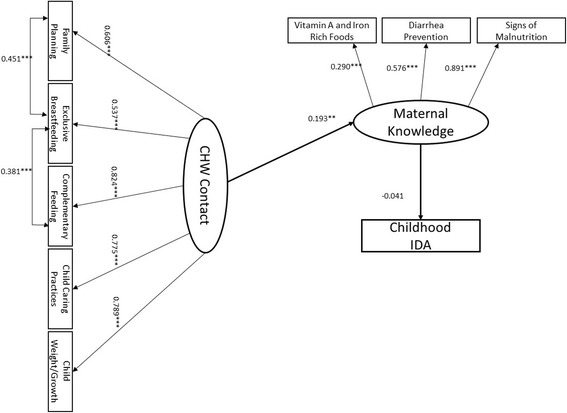
Structural equation model (SEM) with standardized path coefficients (thicker lines) for the effect of Community Health Worker (CHW) contact on Maternal Knowledge and Childhood Iron Deficiency Anemia (IDA). Model is adjusted for household socio-economic status (SES), child’s age less than 24 months, gender, exclusive breastfeeding status, and mother’s anemia status. Significant paths not shown: SES→Childhood IDA (β = 0.076, SE = 0.034, *p* = 0.023); Child Age (<24 months) →Childhood IDA (β = 0.186, SE = 0.032, *p* < .0001); Mother’s IDA→Childhood IDA (β = 0.087, SE = 0.032, *p* = 0.007); Child Age (<24 months) →Maternal Knowledge (β = -0.125, SE = 0.041, *p* = 0.002). Model fit: Number of free parameters = 62; Δ*X* ^2^ = 2.857, Δ*df =* 3, *p* = 0.4142. RMSEA = 0.024, CI: 0.011, 0.035, *p* = 1.000; CFI = 0.972; TLI = 0.959; WRMR = 0.878; *p < .05; **p ≥ .001; ***p < .001

**Fig. 2:**
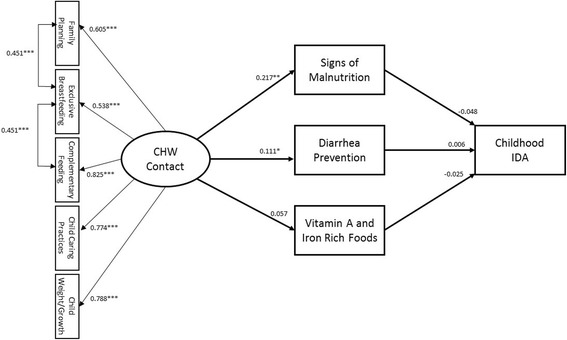
Structural equation model (SEM) with standardized path coefficients for the effect of Community Health Worker (CHW) contact on each of the three Maternal Knowledge categories and Childhood Iron Deficiency Anemia. Model is adjusted for household socio-economic status (SES), child’s age less than 24 months, gender, exclusive breastfeeding status and mother’s anemia status. Significant paths not shown: SES→Diarrhea Prevention (β = -0.093, SE = 0.033, *p* = 0.005); Child Age (<24 months) →Vitamin A and Iron Rich Foods (β = -0.071, SE = 0.035, *p* = 0.042); Child Age (<24 months) →Signs of Malnutrition (β = -0.097, SE = 0.034, *p* = 0.004); Mother’s Anemia→Signs of Malnutrition (β = -0.074, SE = 0.037, *p* = 0.043). Model fit: Number of free parameters = 75; Δ*X* ^2^ = 11.653, Δ*df =* 4, *p* = 0.0201. RMSEA = 0.027, CI: 0.015, 0.038, *p* = 1.000; CFI = 0.969; TLI = 0.946; WRMR = 0.843; *p < .05; **p ≥ .001; ***p < .001

In this study we were interested in testing the hypothesis that contact with CHWs would directly impact maternal knowledge of key child health outcomes while indirectly impacting childhood IDA through increased maternal knowledge. As we modeled CHW contact and maternal knowledge as latent constructs, structural equation modeling (SEM) techniques represented the best statistical approach [25]. To test our study hypothesis, we fitted the proposed path model in Fig. 1 using the two-step approach [26]. In the modeling analysis, child’s age (0–24 months, 25+ months) and gender (boys and girls), child’s exclusive breastfeeding status as reported by the mother and defined as six months of only breastmilk, mother’s anemia status, and socio-economic status (SES), were included as control variables. We used housing characteristics (type of roof, floor materials and number of bedrooms) to create a latent indicator that would be a more reliable proxy of SES [27]. The SEM models were estimated using weighted least square estimator (WLSMV) and theta parameterization on the raw data [28]. WLSMV estimator is especially suitable to the modeling of categorical data as it does not make normality assumptions. To evaluate the effect of CHW on specific maternal knowledge categories, another path model in Fig. 2 was tested using the same modeling approach. The analyses were completed using the software *Mplus*, version 7.11 (Muthen & Muthen, 2013). Data-model fit statistics all met the established cut-off criteria for Root Mean Square Error of Approximation (RMSEA ≤ 0.06), Weighted Root Mean Square Residual (WRMSR ≤ 0.06), Comparative Fit Index (CFI ≤ 1.0), and the Tucker Lewis Index (TLI ≥0.95), respectively. In addition, the non-significant nested model chi-square comparison test showed that estimating the SEM model did not result in a significant decrement in model fit [14]. The complex survey design was accounted for by adjusting for clustering, stratification and applying a finite population adjustment.

## Results

The unweighted and weighted frequency of different characteristics of the sample and the distribution of the variables used in our analyses are summarized in Table 1. Of the 821 participants interviewed, we excluded 207 observations from the analyses; 15 women who were visiting the region during the study, 36 children and 32 mothers with missing hemoglobin measurements and 116 caretakers since they did not complete the maternal health section of the questionnaire and 8 mothers older than 49 years. The final sample recruited for this study consisted of a total of 621 mother and child dyads, 292 boys and 328 girls. We lacked gender information for one child. The prevalence of childhood anemia in the sample was 36.2% (Standard Error = 1.8). Overall, mothers had moderate to high scores (>0.60) on the different knowledge categories. In addition, a large majority reported to have received health promotion education from a CHW.

**Table 1:** Unweighted and weighted distribution of the characteristics of study participants and descriptive statistics of variables used

Indicators (Observations = 621) (Sum of Weights = 1473)	Role	Unweighted Frequency	Weighted % (SE)
Child’s Age	Control variable		
< 24 months		422	68.2 (1.8)
≥ 24 months		196	31.3 (1.7)
Child’s Gender	Control variable		
Boy		292	47.9 (1.8)
Girl		328	52.0 (1.9)
Childhood iron deficiency anemia	Outcome		
No		398	63.8 (1.8)
Yes		223	36.2 (1.8)
Maternal iron deficiency anemia	Control variable		
No		468	73.6 (2.4)
Yes		153	26.4 (2.4)
Child was exclusively breastfed	Control variable		
No		151	23.0 (2.3)
Yes		420	65.6 (7.5)
Roof Material	SES factor indicator		
Corrugated Iron Sheets/Cement		482	77.6 (1.8)
Other		139	22.4 (1.8)
Floor Material	SES factor indicator		
Tile/Cement Floor		305	50.2 (4.1)
Dirt Floor or Other		316	49.8 (4.1)
Number of Bedrooms^a^	SES factor indicator		
≤ Two Rooms		84	13.1 (0.8)
≥ Three Rooms		517	83.5 (0.7)
Knowledge Received from CHW	CHW factor indicators		
Family Planning		398	63.2 (4.3)
Exclusive Breastfeeding		533	85.9 (0.4)
Complementary Feeding		412	67.5 (2.5)
Child Caring Practices		358	58.6 (1.7)
Child Weight/Growth Monitoring		269	43.4 (1.9)
Maternal Mean Knowledge Scores	Knowledge Factor Indicators	Mean (SD)	Mean (SE)
Signs of Malnutrition		0.90 (0.30)	0.84 (0.01)
Diarrhea Prevention		0.84 (0.35)	0.89 (0.01)
Vitamin A and Iron Rich Foods		0.64 (0.42)	0.64 (0.02)

^a^Based on an average household size of 5 for the study region and a minimum of two rooms to sleep for the family. SE = Standard error of weighted frequencies/means; weighted frequencies are rounded to the nearest whole number. SD = Standard deviation

### CHW Contact, Maternal Knowledge and Childhood Anemia

The estimated direct and indirect paths for the effect of CHW contact on childhood anemia as mediated by overall maternal knowledge are presented in Fig. 1. CHW contact was significantly associated with maternal knowledge of key child health practices (β = 0.193, SE = 0.058, *p* = 0.001). However, we observed a negative association between maternal knowledge and child anemia (β = -0.041, SE = 0.044, *p* = 0.354), but the association was not statistically significant at *p* < .05 level. While CHW had a negative indirect association with childhood anemia via maternal knowledge (β = -0.008, SE = 0.009, *p* = 0.382), the path did not reach statistical significance. In this model, significant predictors of child anemia included child’s age less than 24 months (β = 0.186, SE = 0.032, *p* < .0001), household SES (β = 0.076, SE = 0.034, *p* = 0.023) and mother’s anemia (β = 0.087, SE = 0.032, *p* = 0.007). Interestingly, we found that child’s age less than 24 months was negatively associated with maternal knowledge (β = -0.125, SE = 0.041, *p* = 0.002) (Figure S1 in Additional file 2: Appendix B).

### CHW and Association with Specific Maternal Knowledge Categories

Figure 2 shows the association between CHW contact and each of the maternal knowledge categories tested. After adjusting for household SES, child’s age under 24 months, exclusive breastfeeding status and mother’s anemia, CHW contact was significantly associated with maternal knowledge regarding diarrheal prevention (β = 0.111, SE = 0.045, *p* = 0.013) and signs of malnutrition (β = 0.217, SE = 0.071, *p* = 0.002), but the association with knowledge of vitamin A and iron rich foods was not statistically significant at *p* < .05 level (β = 0.057, SE = 0.032, *p* = 0.074). Neither of the knowledge categories resulted in a significant effect on childhood anemia status (Fig. 2). Child’s age less than 24 months was negatively associated with knowledge of vitamin A and iron-rich foods (β = -0.071, SE = 0.035, *p* = 0.042), signs of childhood malnutrition (β = -0.097, SE = 0.034, *p* = 0.004) and childhood anemia (β = 0.186, SE = 0.032, *p* < 0.0001). Household SES was negatively associated with knowledge of diarrheal prevention practice (β = -0.093, SE = 0.033, *p* = 0.005) and positively associated with childhood anemia (β = 0.078, SE = 0.034, *p* = 0.024), while mother’s anemia status was negatively associated with knowledge of malnutrition signs (β = -0.074, SE = 0.037, *p* = 0.043) and positively associated with childhood anemia (β = 0.084, SE = 0.031, *p* = 0.007) (Figure S2 in Additional file 2: Appendix B).

## Discussion

In this study, we assessed the impact of a long standing CHW intervention on childhood anemia in rural Haiti as mediated by maternal knowledge. In our sample, 38.8% of the children had anemia; substantially lower than what is observed in other rural regions of Haiti where the prevalence is estimated to be 64.5% [22]. The overall findings of this study provide evidence that CHW interventions may increase maternal knowledge of key child health practices in rural Haiti. However, this knowledge was not associated with lower childhood anemia prevalence in the study region. Interestingly, we found that lower socio-economic status, as measured by household characteristics, was associated with an increase in anemia prevalence. An important interpretation of this finding would be that household poverty, a key factor for anemia, has not been emphasized in child health intervention. Several studies have demonstrated that independent of maternal education or knowledge improvement, child health is significantly impacted by household socio-economic status [14, 29]. It is likely that, even though mothers are knowledgeable regarding child anemia and its prevention, they lack the economic means to apply acquired knowledge. Our findings stress the fact that in poor communities, interventions targeted at improving childhood nutritional status may have to concurrently impact household-level socio-economic standing.

We also found that mother’s anemia and child’s age < 24 months predicted childhood anemia, which is consistent with the extent literature [23, 30, 31]. These findings reinforce recommendations that interventions to reduce childhood anemia should target children in the first 24 months of life and women of childbearing age [32]. In addition to testing the impact of CHW contact on overall knowledge, we modeled impact on specific knowledge categories. Interestingly, although CHW contact increased knowledge of diarrheal disease prevention and recognition of malnutrition signs in the child, we did not observe a significant association between CHW contact and knowledge of iron and vitamin A rich food in this study. The findings highlight that this area should be emphasized in subsequent *Colvol* trainings and community-based small group discussions in the study region.

There are limitations to our study. First the SEM method makes strong causal assumptions, but data used for these analyses are cross sectional. Second, we elected for a simple model with few variables to explain the impact of the CHW intervention on maternal knowledge and childhood anemia. It is likely that the model did not fully capture the intricacies of the CHW intervention and its impact on child health in the study region. For example, maternal education, an important determinant of child health, especially in resource poor settings [33, 34] was not included in our model as over a quarter of the sample did not provide answers to the education question. Also, we did not include indicators on maternal belief system that may have affected child nutritional practices, such as exclusive breastfeeding [35, 36], as these were not measured. The intervention was implemented uniformly throughout the study region, thus we did not have a comparison group. In addition, due to small sample size limitations, we were not able to compare findings across villages. Such comparisons would have strengthened our findings. When measuring the latent construct, CHW contact, we assumed that all women had similar number of contacts with the CHW in their community. It is possible that some women had more contact then others which would have impacted their level of knowledge. Also, as CHW contact and knowledge categories were based on self-report, misreporting cannot be ruled out. The women would have also received knowledge during their interaction with SBHF community health nurses and hospital staff and we did not account for this additional source of knowledge in our analyses. Nevertheless, it is expected that CHWs will duplicate health literacy messages in the community. The women were asked to report on knowledge gain from a CHW and we do not believe they misreported knowledge acquired during their interaction with SBHF staff as coming from a CHW. We did not account for chronic infection that may impact iron absorption and lead to IDA in young children [32]. Finally, the question used to test maternal knowledge of diarrheal disease prevention practices included answer choices such as “*use sanitary latrines”*, “*use safe fluids”* (Additional file 1: Appendix A) and many of the women did not mention these options. It is likely that poorer mothers with limited access would not have reported these items on the questionnaire; this omission impacted their knowledge score. Future analyses should explore methods to capture knowledge that is not correlated with participants’ SES.

## Conclusions

The involvement of CHWs in health care delivery in resource poor settings is well known and several studies have documented positive effect on maternal and child health [16, 37]. In developing countries such as Haiti with critical shortage of health professionals, CHWs are essential components of primary health care delivery to rural and isolated communities [16]. As members of the communities they serve, CHWs are especially equipped to deliver health advocacy messages in the form of small group discussions. We show that using such a model may result in a significant increase in maternal knowledge of key child health practices. However, CHW interventions should be adapted to the local context and what works in one setting may not provide the same results in another [17]. In our study region, CHWs have an impact on maternal knowledge; however, there exists a knowledge-action gap potentially driven by household poverty.

## Additional files

Additional file 1: Appendix A. Questionnaire items used in this study. (DOCX 17 kb)

Additional file 2: Appendix B. Full models showing all significant paths. (DOCX 151 kb)

## Abbreviations

CFI: Comparative Fit Index
CHW: Community Health Workers
EBF: Exclusive Breastfeeding
ID: Iron Deficiency
IDA: Iron Deficiency Anemia
MCH: Maternal Child Health
RMSEA: Root Mean Square Error of Approximation
SBHF: St. Boniface Haiti Foundation
SEM: Structural Equation Modeling
SES: Socio-economic Status
TLI: Tucker Lewis Index
UNICEF: United Nations Children’s Emergency Fund
WRMSR: Weighted Root Mean Square Residual

## References

[1] World Health Organization. The global prevalence of anaemia in 2011 [Internet]. WHO. 2015 [cited 2015 Jul 24]. Available from: http://www.who.int/nutrition/publications/micronutrients/global_prevalence_anaemia_2011/en/.

[2] Miller JL. Iron Deficiency Anemia: A Common and Curable Disease. Cold Spring Harb Perspect Med [Internet]. 2013 Jul [cited 2015 Jul 14];3(7). Available from: http://www.ncbi.nlm.nih.gov/pmc/articles/PMC3685880/10.1101/cshperspect.a011866 PMC3685880 23613366

[3] WHO | Children: reducing mortality [Internet]. WHO. [cited 2014 Dec 9]. Available from: http://www.who.int/mediacentre/factsheets/fs178/en/.

[4] Stoltzfus RJ (2001). Summary: implications for research and programs. J Nutr.

[5] Saloojee H, Pettifor JM (2001). Iron deficiency and impaired child development. BMJ.

[6] 2014 Report - Countdown to 2015 [Internet]. [cited 2014 Dec 10]. Available from: http://www.countdown2015mnch.org/reports-and-articles/previous-reports/2014-report.

[7] LeVine RA, Rowe ML (2009). Maternal literacy and child health in less-developed countries: evidence, processes, and limitations. J Dev Behav Pediatr.

[8] Abuya BA, Ciera J, Kimani-Murage E (2012). Effect of mother’s education on child’s nutritional status in the slums of Nairobi. BMC Pediatr.

[9] Desai S, Alva S (1998). Maternal education and child health: is there a strong causal relationship?. Demography.

[10] Gakidou E, Cowling K, Lozano R, Murray CJ (2010). Increased educational attainment and its effect on child mortality in 175 countries between 1970 and 2009: a systematic analysis. Lancet.

[11] Armar-Klemesu M, Ruel MT, Maxwell DG, Levin CE, Morris SS (2000). Poor maternal schooling is the main constraint to good child care practices in Accra. J Nutr.

[12] Glewwe P (1999). Why does mother’s schooling raise child health in developing countries? Evidence from Morocco. J Hum Resour.

[13] Ruel MT, Levin CE, Armar-Klemesu M, Maxwell D, Morris SS (1999). Good care practices can mitigate the negative effects of poverty and low maternal schooling on children’s nutritional status: evidence from Accra. World Dev.

[14] Ruel MT, Habicht JP, Pinstrup-Andersen P, Gröhn Y (1992). The mediating effect of maternal nutrition knowledge on the association between maternal schooling and child nutritional status in Lesotho. Am J Epidemiol.

[15] Bhutta ZA, Ahmed T, Black RE, Cousens S, Dewey K, Giugliani E (2008). What works? Interventions for maternal and child undernutrition and survival. Lancet.

[16] Lehmann U, Sanders D. Community Health Workers: What Do We Know About Them? The State of the Evidence on Programmes, Activities, Costs an Impact on Health Outcomes of Using Community Health Workers [Internet]. 2007 Jan [cited 2013 Mar 21]. Available from: http://www.hrhresourcecenter.org/node/1587.

[17] Gilmore B, McAuliffe E (2013). Effectiveness of community health workers delivering preventive interventions for maternal and child health in low- and middle-income countries: a systematic review. BMC Public Health.

[18] Lewin S, Munabi-Babigumira S, Glenton C, Daniels K, Bosch-Capblanch X, van Wyk BE (2010). Lay health workers in primary and community health care for maternal and child health and the management of infectious diseases. Cochrane Database Syst Rev Online.

[19] WHO | Using lay health workers to improve access to key maternal and newborn health interventions in sexual and reproductive health [Internet]. WHO. [cited 2014 Apr 29]. Available from: http://www.who.int/reproductivehealth/publications/maternal_perinatal_health/en/.

[20] Jerome JG, Ivers LC (2010). Community health workers in health systems strengthening: a qualitative evaluation from rural Haiti. AIDS Lond Engl.

[21] Perry HB, King-Schultz LW, Aftab AS, Bryant JH (2007). Health equity issues at the local level: Socio-geography, access, and health outcomes in the service area of the Hôpital Albert Schweitzer-Haiti. Int J Equity Health.

[22] Cayemittes M, Busangu, Michelle Fatuma, Bizimana, Jean de Dieu, Barrere, Bernard, Severe, Blaise, Cayemittes, Viviane, et al. Enquête Mortalité, Morbidité et Utilisation des Services (EMMUS-V), Haiti, 2012 [Internet]. Calverton, Maryland, USA: MSPP, IHE et ICF International; 2013 [cited 2015 Jul 25]. Available from: http://mspp.gouv.ht/site/downloads/EMMUS%20V%20document%20final.pdf.

[23] Ayoya MA, Ngnie-Teta I, Séraphin MN, Mamadoultaibou A, Boldon E, Saint-Fleur JE (2013). Prevalence and risk factors of anemia among children 6-59 months old in Haiti. Anemia.

[24] Séraphin MN, Ngnie-Teta I, Ayoya MA, Khan MR, Striley CW, Boldon E (2014). Determinants of institutional delivery among women of childbearing age in Rural Haiti. Matern Child Health J.

[25] Gunzler D, Chen T, Wu P, Zhang H (2013). Introduction to mediation analysis with structural equation modeling. Shanghai Arch Psychiatry.

[26] Anderson JC, Gerbing DW (1988). Structural equation modeling in practice: A review and recommended two-step approach. Psychol Bull.

[27] Balen J, McManus DP, Li Y-S, Zhao Z-Y, Yuan L-P, Utzinger J (2010). Comparison of two approaches for measuring household wealth via an asset-based index in rural and peri-urban settings of Hunan province, China. Emerg Themes Epidemiol.

[28] Asparouhov T, Muthén B (2010). Weighted Least Squares Estimation with Missing Data.

[29] Saaka M (2014). Relationship between mothers’ nutritional knowledge in childcare practices and the growth of children living in impoverished rural communities. J Health Popul Nutr.

[30] Stoltzfus RJ (2008). Research needed to strengthen science and programs for the control of iron deficiency and its consequences in young children. J Nutr.

[31] Heidkamp RA, Ngnie-Teta I, Ayoya MAA, Stoltzfus RJ, Mamadoultaibou A, Durandisse EB (2013). Predictors of anemia among haitian children aged 6 to 59 months and women of childbearing age and their implications for programming. Food Nutr Bull.

[32] Lutter CK (2008). Iron deficiency in young children in low-income countries and new approaches for its prevention. J Nutr.

[33] Mangrio E, Hansen K, Lindström M, Köhler M, Rosvall M (2011). Maternal educational level, parental preventive behavior, risk behavior, social support and medical care consumption in 8-month-old children in Malmö, Sweden. BMC Public Health.

[34] Imdad A, Yakoob MY, Bhutta ZA (2011). Impact of maternal education about complementary feeding and provision of complementary foods on child growth in developing countries. BMC Public Health.

[35] Walingo MK, Mutuli LA (2014). Influence of maternal beliefs, attitude, perceived behavior on breast-feeding among post partum mothers in Western Kenya. Pak J Nutr.

[36] Osman H, El Zein L, Wick L (2009). Cultural beliefs that may discourage breastfeeding among Lebanese women: a qualitative analysis. Int Breastfeed J.

[37] Winch PJ, Gilroy KE, Wolfheim C, Starbuck ES, Young MW, Walker LD (2005). Intervention models for the management of children with signs of pneumonia or malaria by community health workers. Health Policy Plan.

