# Supplementary material for: Childhood anemia in Rural Haiti: the potential role of community health workers
Source: Glob Health Res Policy. 2017 Jan 23;2:3. doi: 10.1186/s41256-016-0022-7 (PMC5683206; doi:10.1186/s41256-016-0022-7)
Supplement: Supplementary file 1 — Questionnaire items used in this study. (DOCX 17 kb) [file 41256_2016_22_MOESM1_ESM.docx]

**Additional file 1 Appendix A**: **Questionnaire Items Used in This Study**

| **maternal and newborn health module** | | | | | **MN** | |
| --- | --- | --- | --- | --- | --- | --- |
| MN35 | DID YOU RECEIVE THE FOLLOWING NUTRITION INFORMATION FROM CHW? |  | NO | YES | |  |
|  |  | On child weight/growth | 0 | 1 | |  |
|  |  | On Exclusive Breast Feeding | 0 | 1 | |  |
|  |  | On Complementary Feeding | 0 | 1 | |  |
|  |  | On Family planning | 0 | 1 | |  |
|  |  | On child caring practices | 0 | 1 | |  |
|  |  | ____________________________________Others= 2 | | | |  |
| MN21 | CAN YOU LIST FOODS THAT ARE RICH IN VITAMIN A?  *(Don’t read choices, record all items mentioned)* | Teff= 1  Wheat = 2  Barley= 3  Leafy vegetables= 4  Orange Fruits and Vegetables= 5  Other Vegetables= 6  Fish= 7  Meat = 8  Eggs = 9  Milk= 10  Other fruits = 11  Oil/butter= 12  Salt = 13  __________________________Other (specify)= 14  DK= 98 | | | |  |
| MN22 | CAN YOU LIST FOODS THAT ARE RICH IN IRON?  *(Don’t read choices, record all items mentioned)* | Teff= 1  Wheat = 2  Barley= 3  Leafy vegetables= 4  Orange Fruits and Vegetables= 5  Other Vegetables= 6  Fish= 7  Meat = 8  Eggs = 9  Milk= 10  Fruits = 11  Oil/butter= 12  Salt = 13  __________________________Other (specify)= 14  DK= 98 | | | |  |
| MN24 | HOW CAN DIARRHOEA BE PREVENTED?  *(Don’t read choices, record all items mentioned)* | Use of safe foods= 1  Use of safe fluids= 2  Washing hands before taking foods= 3  Washing hands with soap after defecation= 4  Washing hand with ash/mud after defecation= 5  Using sanitary latrine= 6  Continue breastfeeding up to 2 years= 7  Proper immunization= 8  _____________________________Other (specify) = 9  Don’t know/not sure= 98 | | | |  |
| MN34 | CAN YOU NAME THE SIGNS THAT A CHILD with malnutrition may have?  *(Don’t read choices, record all items mentioned)* | Thin= 1  Short= 2  Old man face (monkey face= 3  Irritable= 4  Change of hair color= 5  Sunken eye ball= 6  Leg edema= 7  __________________________Other (specify)= 7  DK= 98 | | | |  |
