# Supplementary material for: Childhood anemia in Rural Haiti: the potential role of community health workers
Source: Glob Health Res Policy. 2017 Jan 23;2:3. doi: 10.1186/s41256-016-0022-7 (PMC5683206; doi:10.1186/s41256-016-0022-7)
Supplement: Supplementary file 2 — Full models showing all significant paths. (DOCX 151 kb) [file 41256_2016_22_MOESM2_ESM.docx]

**Additional file 2: Appendix B.** Full models showing all significant paths.


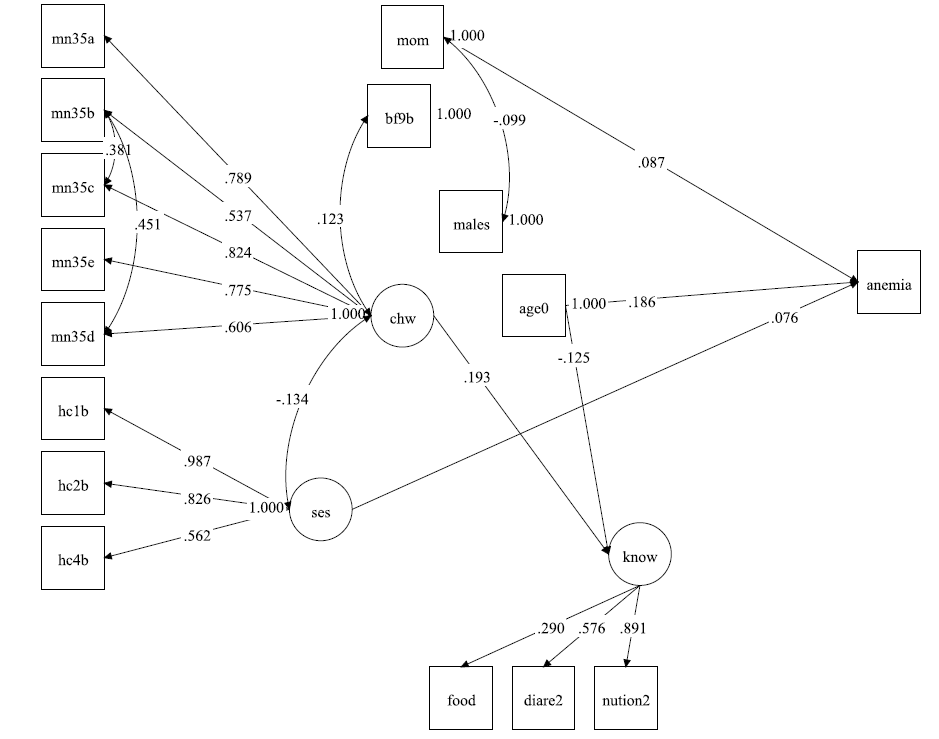


**Figure S1: Structural equation model (SEM) with standardized path coefficients for the effect of Community Health Worker (CHW) contact on Maternal Knowledge and Childhood Iron Deficiency Anemia (IDA)**. Model is adjusted for household socio-economic status (SES), child’s age less than 24 months (age0), gender (males), exclusive breastfeeding status (bf9b) and mother’s anemia status (mom). All statistically significant paths are shown. **Model fit**: Number of free parameters=62; Δ*X*^2^=2.857, Δ*df=*3, p=0.4142. RMSEA=0.024, CI: 0.011, 0.035, p=1.000; CFI=0.972; TLI=0.959; WRMR=0.878; *p<.05; **p≥.001; ***p<.001.


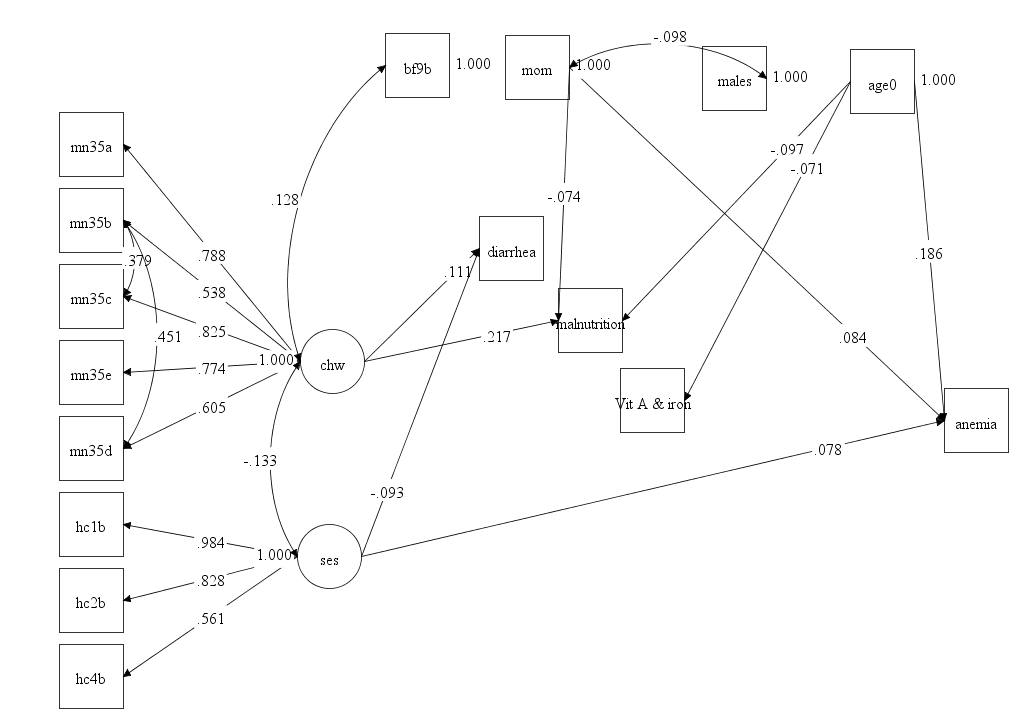


**Figure S2: Structural equation model (SEM) with standardized path coefficients for the effect of Community Health Worker (CHW) contact on each of the three Maternal Knowledge categories and Childhood Iron Deficiency Anemia**. Model is adjusted for household socio-economic status (SES), child’s age less than 24 months (age0), gender (males), exclusive breastfeeding status (bf9b) and mother’s anemia status (mom). All statistically significant paths are shown. **Model fit**: Number of free parameters=75; Δ*X*^2^=11.653, Δ*df=*4, p=0.0201. RMSEA=0.027, CI: 0.015, 0.038, p=1.000; CFI=0.969; TLI=0.946; WRMR=0.843; *p<.05; **p≥.001; ***p<.001.
